# Effect of preservation versus sacrifice of the posterior cruciate ligament on clinical outcomes after medial congruent total knee arthroplasty using mechanical alignment: A systematic review and meta‐analysis

**DOI:** 10.1002/jeo2.70873

**Published:** 2026-08-03

**Authors:** Mansour Bahardoust, Mehdi Mohammadpour, Habib Gorgani, Meisam Haghmoradi, Sajad Fakoor

**Affiliations:** ^1^ Breast Cancer Research Center Iran University of Medical Sciences Tehran Iran; ^2^ Department of Orthopedics, Bone and Joint Reconstruction Research Center, School of Medicine Iran University of Medical Sciences Tehran Iran; ^3^ Department of Orthopedic Surgery, Faculty of Medicine Golestan University of Medical Sciences Golestan Iran; ^4^ Department of Orthopedic Surgery Urmia University of Medical Sciences Urmia Iran; ^5^ Department of Orthopedics and Traumatology, Faculty of Medicine Ahvaz Jundishapur University of Medical Sciences Ahvaz Iran

**Keywords:** functional outcome, medial congruent prosthesis, posterior cruciate ligament preservation, surgery, total knee arthroplasty

## Abstract

**Purpose:**

The debate on whether to preserve or sacrifice the posterior cruciate ligament (PCL) during medial congruent (MC) total knee arthroplasty (TKA) is still ongoing. Currently, there is no comprehensive consensus due to the limited evidence on which approach yields better outcomes. This systematic review and meta‐analysis aimed to compared the functional outcomes of PCL retaining (PR) versus PCL sacrificing (PS) in MC TKA.

**Methods:**

Two independent researchers to find relevant articles, with no time limit until 4 August 2025, searched PubMed/Medline, Scopus, Web of Science and Embase databases. Postoperative functional outcomes were compared between the PR and PS groups, in accordance with preferred reporting items for systematic reviews and meta‐analyses guidelines. The Cochran *Q* and *I* ^2^ tests were employed to assess heterogeneity between studies.

**Results:**

Six cohort studies, including 903 patients, were included. The pooled estimate of the four studies demonstrated that at 3 months after surgery, the mean difference in postoperative KSS score between the PR and PS groups was 1.15, which was not statistically significant (95% CI: –1.11, 3.31). At 12 months after surgery, the mean Knee Society Score (KSS) reached statistical significance in favour of the PR group (MD: 1.97, *p* < 0.05); however, this difference did not exceed the minimal clinically important difference (MCID) of 5.3 points, indicating a lack of clinical significance There was no significant difference in the mean Oxford Knee Score (OKS) score at 3 and 12 months after surgery between the PR and PS groups.

**Conclusions:**

There appears to be no clinically meaningful difference in PR or PS during MC TKA in improving short‐term clinical outcomes based on moderate‐certainty evidence (Level IV). In the long term, preserving the PCL may lead to greater improvements in physical function compared to losing the PCL. However, no significant differences were noted in other clinical or functional outcomes, nor in complication rates, between the two groups.

**Level of Evidence:**

Level III.

**Abbreviations**
CPAK classification: coronal plane alignment of the knee classification
DVT: deep vein thrombosis
FA: functional alignment
FJS: forgotten joint score
KA: kinematic alignment
KSS: Knee society score
MA: mechanical alignment
MC: medial congruent
MP: medial‐pivot
OKS: Oxford knee score
PCL: posterior cruciate ligament
ROM: range of motion
TKA: total knee arthroplasty
WOMAC: The Western Ontario and McMaster Universities Arthritis Index

## INTRODUCTION

Total knee arthroplasty (TKA) is among the most frequently performed orthopaedic surgeries worldwide for managing end‐stage knee osteoarthritis, and its incidence continues to grow as the population ages. Recent studies have demonstrated that TKA is associated with significant improvements in clinical and functional outcomes, primarily due to advancements in implant design, the utilisation of advanced prosthetic materials and the adoption of innovative surgical techniques [19, 31]. Research indicates that implants designed to replicate natural knee kinematics can lead to greater improvements in patient satisfaction and overall results after TKA [12, 27]. The posterior stabilised (PS) design mimics the function of the posterior cruciate ligament (PCL), allowing for femoral retraction during deep knee flexion [43]. This type of implant offers both advantages and disadvantages [42, 44, 50]. In recent years, various implants have been introduced for TKA [2, 26, 51]. One of these is the medial congruent (MC) TKA implant. This implant is a type of medial‐pivot (MP) TKA that maintains a consistent polyethylene design, regardless of the status of the PCL [7].

The MC implant distinguishes itself from traditional MP implants by incorporating a highly asymmetric polyethylene component, where the curvature of the medial compartment of the polyethylene liner is more cupped and congruent with the medial femoral condyle. This design closely mimics natural knee anatomy, promoting consistent internal kinematics and lateral movement during flexion [9, 12, 53]. This design aims to enhance anteromedial stability while permitting internal rotation throughout the knee's range of motion (ROM). Notably, the anterior edge of the polyethylene enhances stability by providing a restraint to posterior translation. This feature enables the sacrifice of the PCL without requiring a post‐CAM mechanism [4, 20, 38, 45, 68].

One of the main and distinctive advantages of the MC prosthesis is increased anteroposterior stability, which reduces the risk of anterolateral pain after TKA, thereby improving operative outcomes and patient satisfaction [9, 24]. In a study by Nishio et al. [45], functional activity and patient satisfaction were better after surgery with the MP prosthesis. Batra et al. [4] also confirmed these results in their studies. They demonstrated that the MC design was associated with favourable clinical and radiological outcomes, which they attributed to its ability to replicate the biomechanics of the natural knee better. Several studies have shown that maintaining the patellofemoral ‘third space’ can be associated with improved functional outcomes and patient satisfaction after MC TKA [1, 38].

In recent years, it has become clear that, in addition to prosthetic design, other factors such as the role of new technologies, especially in young surgeons to assess PROMs, as well as robotic assistance with postoperative clinical outcomes, have become apparent [23, 48]. Numerous studies have demonstrated improved clinical outcomes and kinematic performance with the mobile‐bearing MC TKA [21, 27, 58]. However, the debate over whether to retain or sacrifice the PCL during MC TKA continues [10]. Research findings on this topic have varied, often due to the use of small sample sizes [10, 21, 58].

As a result, the optimal management of the PCL in conjunction with the MC prosthesis during TKA remains controversial. There is no consensus among knee surgeons on whether preserving or sacrificing the PCL leads to better outcomes. In this systematic review and meta‐analysis, we examined the functional consequences and postoperative complications in patients who underwent MC TKA using mechanical alignment (MA), comparing those with PCL retaining (PR) to PCL sacrificing (PS).

## METHODS

In this systematic review, we assessed all observational studies examining clinical and functional outcomes, as well as complication rates, following MC TKA using MA with or without PR. Our review adhered to the preferred reporting items for systematic reviews and meta‐analyses 2020 guideline [49] (Supporting Information: Table S1), and it is registered with PROSPERO (CRD420251118760). We categorised the patient cohort into two distinct groups based on PCL status: PR and PS. The review includes exclusively retrospective cohort studies and no randomised controlled trials; therefore, the overall level of evidence is Level IV.

### Methods for literature search

Two independent researchers without time limits searched the PubMed/Medline, Embase, Scopus, Google Scholar and Web of Science databases until 4 August 2025. The search strategy is reported in the supplement (Supporting Information: Table S2).

### Inclusion and exclusion criteria

This meta‐analysis focuses on observational studies cohort, case‐control and cross‐sectional—that assessed the impact of PR versus PS in patients undergoing MC TKA using MA. Inclusion criteria specified that studies report either the mean difference between the preservation and sacrifice groups (PR/PS) or the mean functional assessment scores, specifically the PCL during MC TKA PR versus PS Knee Society score (KSS), Oxford knee score (OKS) and forgotten joint score (FJS) Studies with at least 3 months of follow‐up after surgery (Minimum time required to produce an evaluable effect based on functional assessment scores after TKA) and a sample size of at least 20 participants in comparative subgroups to ensure sufficient precision for pooled estimates. Previous studies have reported minimum minimal clinically important differences (MCIDs) for KSS and OKAS of 5.3 and 4.9 points, respectively [28, 36].

Exclusion criteria included studies focusing on the effects of different prosthetic types on functional outcomes without specific reporting by prosthesis type, as well as reviews, meta‐analyses, editorials, experimental studies, case reports, studies lacking full‐text access and those published in non‐English‐language journals.

The primary outcomes evaluated included functional outcomes, as determined by the KSS, OKS and FJS instruments, while secondary outcomes included complication rates and range‐of‐motion assessments.

### Study selection

**Figure 1:**
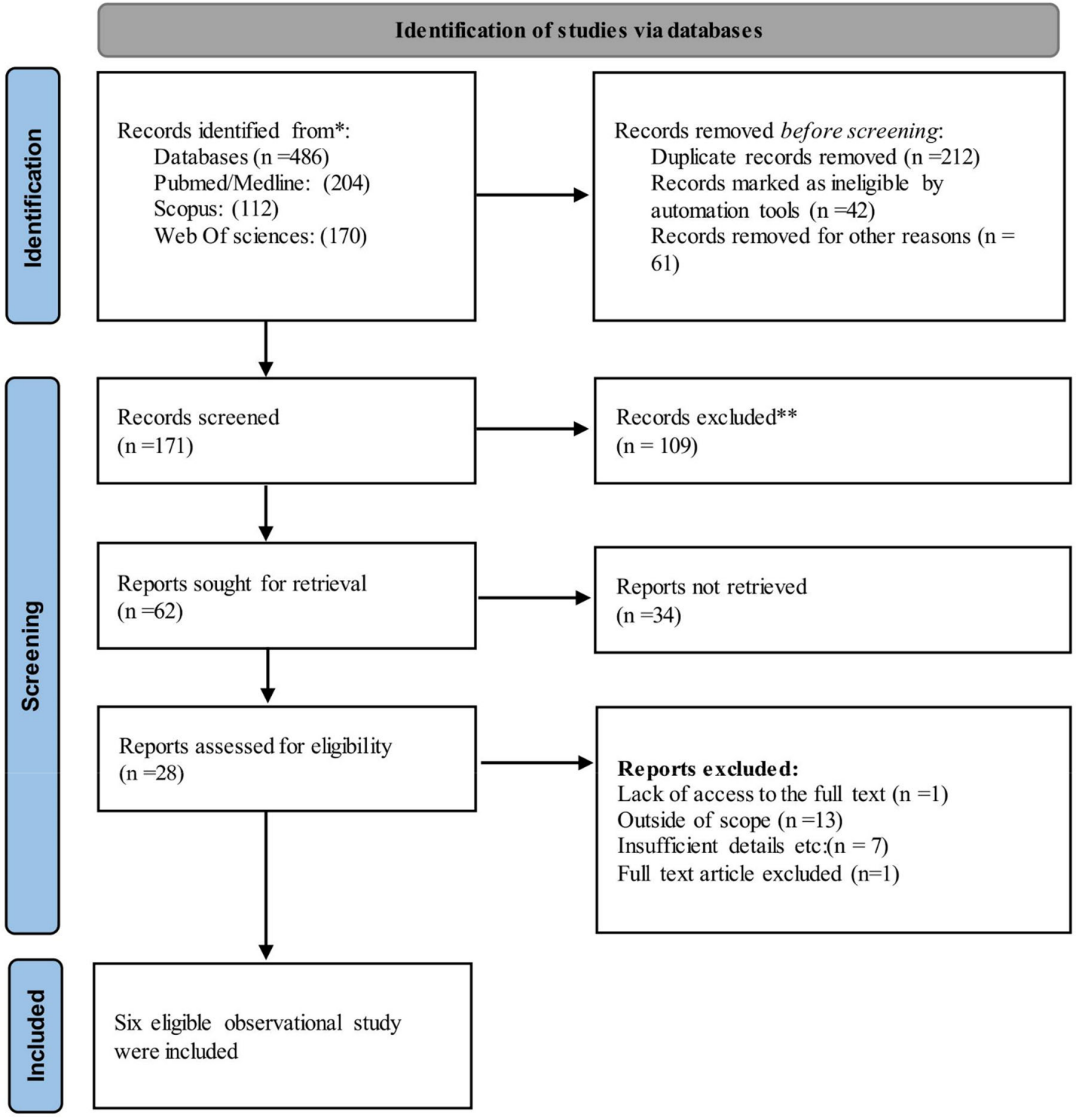
Flowchart page of studies based on preferred reporting items for systematic reviews and meta‐analyses 2020. *Articles found from all databases. **Articles excluded due to lack of sufficient details.

In our initial search of the PubMed/Medline, Embase, Scopus and Web of Science databases, we identified 486 studies pertinent to our research question using established search strategies. We employed EndNote 22 software (Baarcompany) to identify and eliminate duplicate entries across databases. Two independent researchers assessed the remaining studies based on their relevance to the research question and their titles and abstracts. A third researcher adjudicated discrepancies in article selection. Ultimately, after applying our defined inclusion and exclusion criteria, we conducted a comprehensive review of the full texts of 28 articles. Based on this review, we included six cohort studies in the subsequent meta‐analysis (Figure 1).

### Data extraction

In this systematic review, we identified essential variables through a comprehensive literature assessment conducted by an orthopaedic knee surgeon and an epidemiologist. An Excel spreadsheet was subsequently created to facilitate the extraction of these variables. To ensure accuracy, two independent reviewers carried out the variable extraction, with a third reviewer addressing any discrepancies that arose.

The data collected encompassed a range of critical details, including the authorship and year of each study, geographical location, study design, mean participant age, gender distribution, verage body mass index (BMI), total patient cohort and the number of patients segmented by surgical procedure. We also documented the incidence of complications for each surgical group and the average outcomes, measured by the OKS, KSS and FJS, for each participant group. Additionally, we recorded the average ROM and the mean duration of follow‐up in years. Whenever feasible, we contacted the authors of the studies to fill any gaps in the data.

### Quality assessment

The quality of the cohort studies included in this review was evaluated utilising the Newcastle‐Ottawa Quality Assessment Scale for Cohort Studies. This method involves a detailed checklist that allocates points across three key domains: Selection, Comparability and Outcome/Exposure. After assessing the studies, the points are totalled, allowing for classification of overall study quality into three categories: poor (0–3 points), fair (4–6 points) and good (7–9 points). Furthermore, we assessed the certainty of the evidence applying the grading of recommendations assessment, development and evaluation (GRADE) approach [52], which provides a structured framework for evaluating the quality and reliability of clinical study results.

### Statistical analyses

Data were analysed utilising Stata software (company: StataCorp) version 17, employing random effects models to control for the effect of study sample size. We used the restricted maximum likelihood (REML) estimator to calculate the between‐study variance (*τ* ^2^) because it gives less biased results than the DerSimonian‐Laird estimator, especially when there are few studies. The choice of heterogeneity estimator can strongly affect pooled effect estimates, prediction intervals and statistical significance. Simulation studies show that different estimators can lead to very different variance estimates in the same meta‐analysis. Since it is not usually best to rely on just one estimator, we also ran sensitivity analyses with other estimators (DerSimonian‐Laird, Paule‐Mandel and Sidik‐Jonkman) to check how robust our results are [54].

The primary effect size, represented by the mean difference in postoperative functional indices between the two cohorts (PS and PR), was reported alongside 95% confidence intervals. Heterogeneity and variance between studies were assessed with *I* ^2^ and *τ* ^2^ values *I* ^2^ interpreted according to the following thresholds: 0%–40% indicating not be important heterogeneity, 30%–60% moderate heterogeneity, 50%–90% substantial heterogeneity and 75%–100% considerable heterogeneity [22, 59].

The assessment for publication bias was conducted using Egger's test, and the findings were illustrated via a funnel plot. Descriptive elements were incorporated through the use of tables and figures. Given the absence of publication bias across the evaluated outcomes, it was deemed unnecessary to implement the Trim and Fill method. Subgroup analyses were stratified according to follow‐up periods of 3 and 12 months postsurgery, while sensitivity analyses were executed to gauge the impact of individual studies on the aggregate outcomes.

## RESULTS

### Study characteristics

A total of six retrospective cohort studies [14, 21, 27, 37, 58] were analysed, encompassing 903 patients who underwent MC TKA, with 516 in the PS group and 387 in the PR group. The mean age of patients at the time of surgery was 68.6 years. Thirty‐two per cent of the PR group and 38% of the PS group were male. The average BMI was 28.1 kg/m^2^. Notably, four studies featured Asian populations, including three conducted in Singapore. Most patients were classified as American Society of Anesthesiologists (ASA) II, with no instances of ASA IV reported. The median follow‐up duration was 12 months, with a range from 3 to 72 years. The quality and certainty of the evidence are reported in Supporting Information: Tables S3 and S4, respectively. The characteristics of patients in the studies included in this systematic review are presented in Table 1.

**Table 1:** Characteristics of patients in studies and quality of included studies.

Author	Country	Study design	Sample size	PR/PS	Sex (female) (PR/PS)	BMI (PR/PS)	ASA classification	Mean follow‐up (Month)	Mean age (PR/PS)	Quality of studies	Certainty of evidence
L Chen [10]	China	Retrospective cohorts	105	76/29	26	25.6	NA	18	69.1	Fair	Low
KMS Khoo [27]	Singapore	Retrospective cohorts	89	44/45	30/31	26.1/27	I:1,II:71, III:17	12	70.6/66.1	Fair	Low
SMP Rossi [58]	Italy	Retrospective cohorts	165	80/85	61/63	28.5/27.9	NA	72	68.6	Good	Moderate
WC Lee [37]	Singapore	Retrospective cohorts	70	26/44	30/20	26.4/29	I:2,II:49, III:19	12	70/66	Good	Moderate
BCM Foong [14]	Singapore	Retrospective cohorts	76	26/50	22/36	29/28	NA	14	67/66	Fair	Moderate
GN Guild III [21]	USA	Retrospective cohorts	398	264/134	158/68	29.7/30.6	I:2,II:279, III:117,	3	70.5/69.1	Good	Moderate

Abbreviations: ASA, American Society of Anesthesiologists; BMI, body mass index; PR, PCL retaining; PS, PCL sacrificing.

#### Functional outcomes

##### KSS

**Figure 2:**
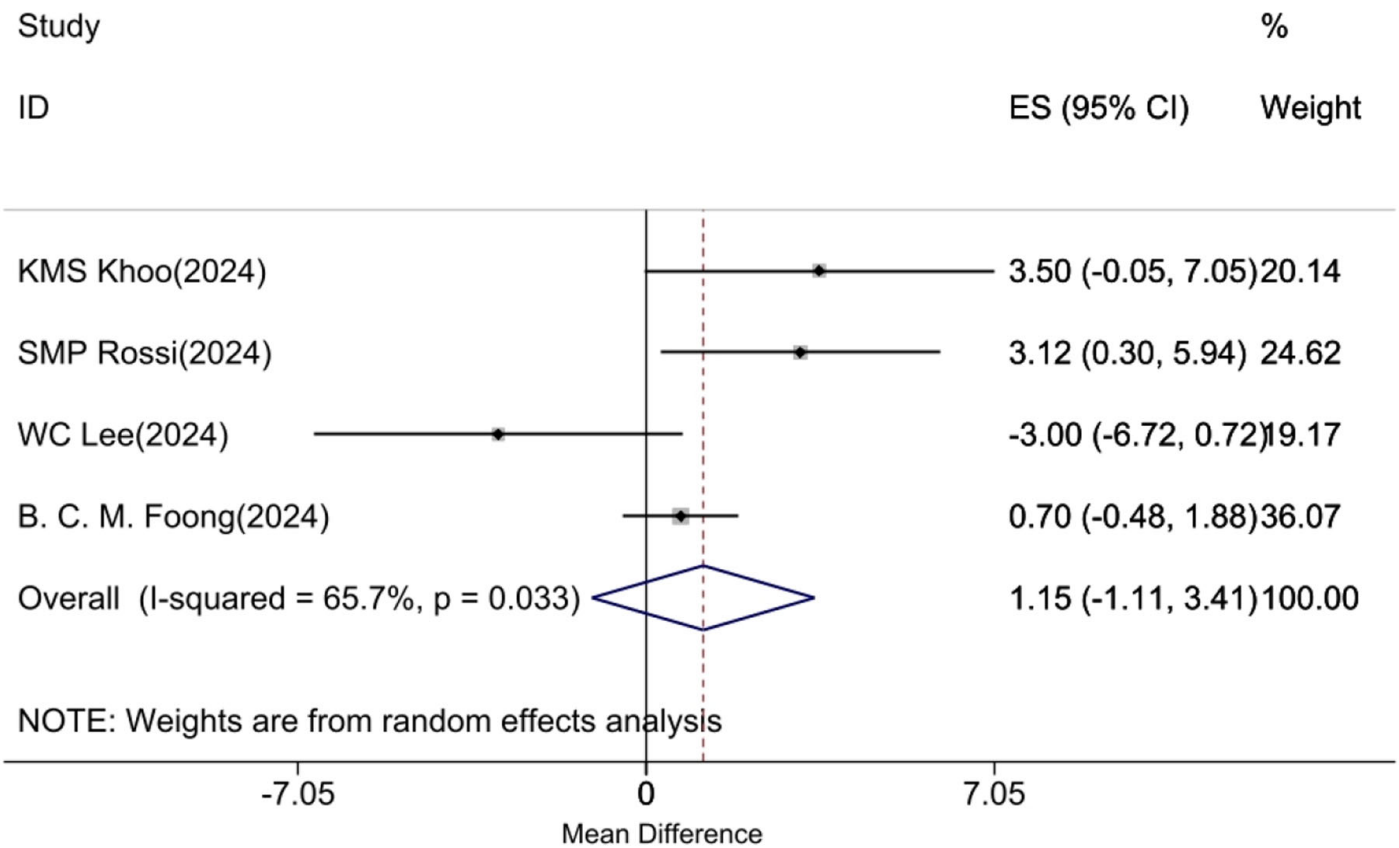
Difference in mean postoperative KSS score between PR and PS groups after 3 months. CI, confidence intervals; KSS, Knee society score; PR, PCL retaining; PS, PCL sacrificing.

**Figure 3:**
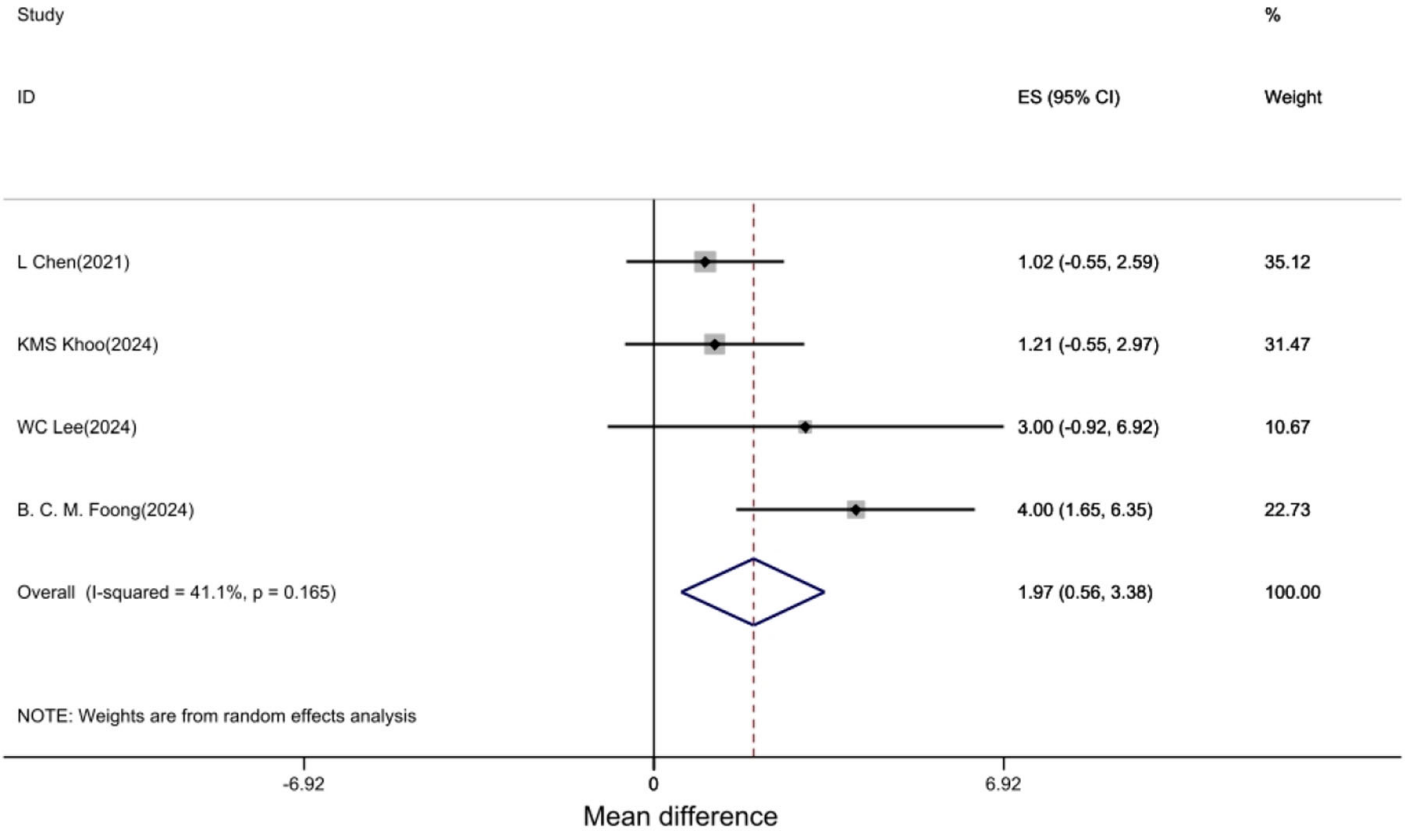
Difference in mean postoperative KSS score between PR and PS groups after 12 months. CI, confidence intervals; KSS, Knee society score; PR, PCL retaining; PS, PCL sacrificing.

The impact of PR versus PS on KSS was evaluated at 3 and 12 months postoperatively across four studies [14, 27, 37, 58]. At 3 months, the pooled mean difference in KSS between the PR and PS groups was 1.15 (95% CI: –1.11 to 3.31, *I* ^2^ = 65.7%, *τ* ^2 ^= 0.81), which was not statistically significant (Figure 2). At the 12‐month mark, the PR group demonstrated a significantly higher mean KSS score than the PS group (Mean Difference: 1.97; 95% CI: 0.56–3.38, *I* ^2^ = 41%, *τ* ^2 ^= 0.54) (Figure 3). Although the difference was statistically significant, it remained below the established MCID threshold of 5.3 points for the KSS. Therefore, this finding does not represent a clinically meaningful improvement.

##### OKS

**Figure 4:**
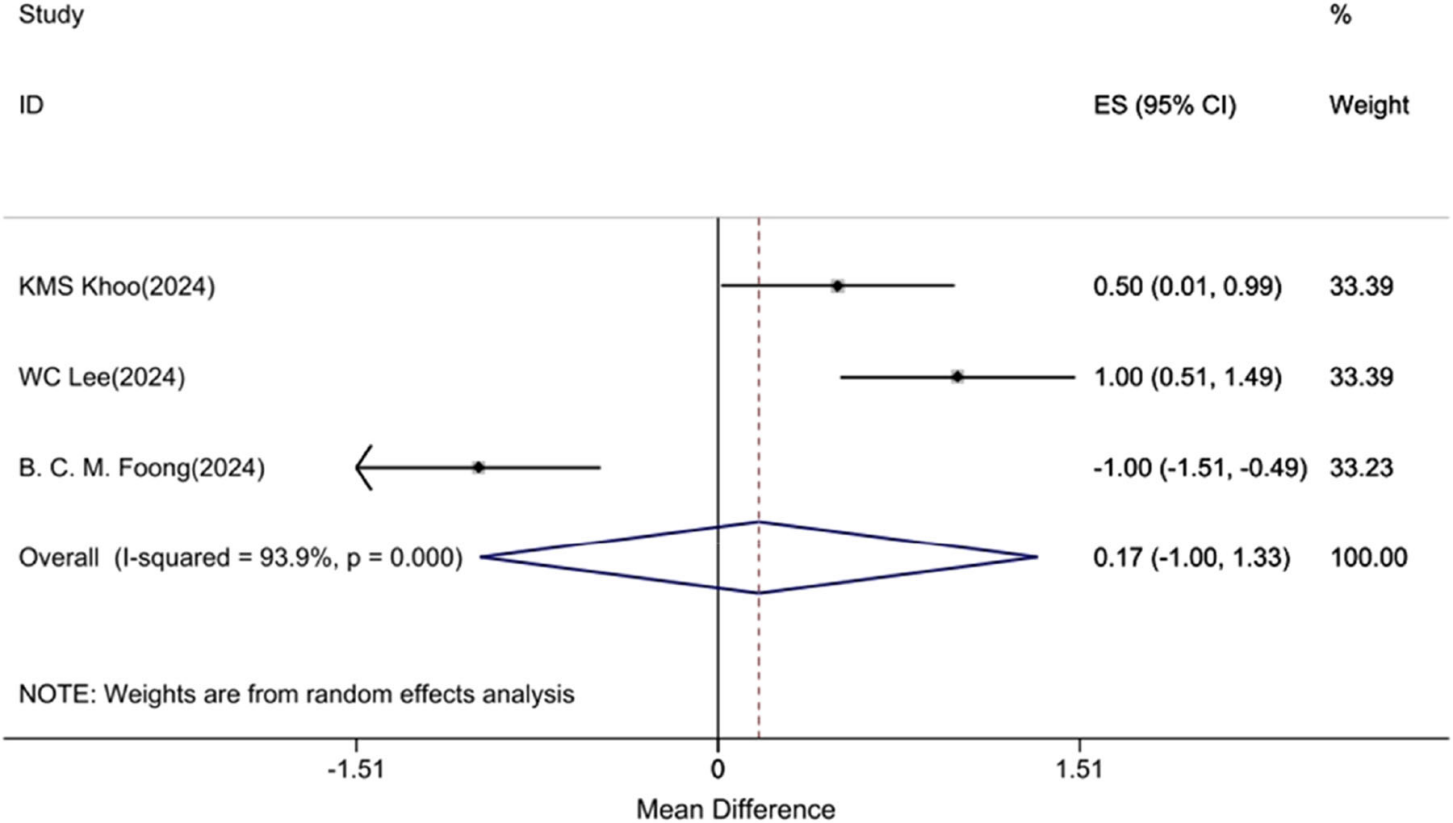
Difference in mean postoperative OKS between PR and PS groups after 3 months. CI, confidence intervals; OKS, Oxford Knee Score; PR, PCL retaining; PS, PCL sacrificing.

**Figure 5:**
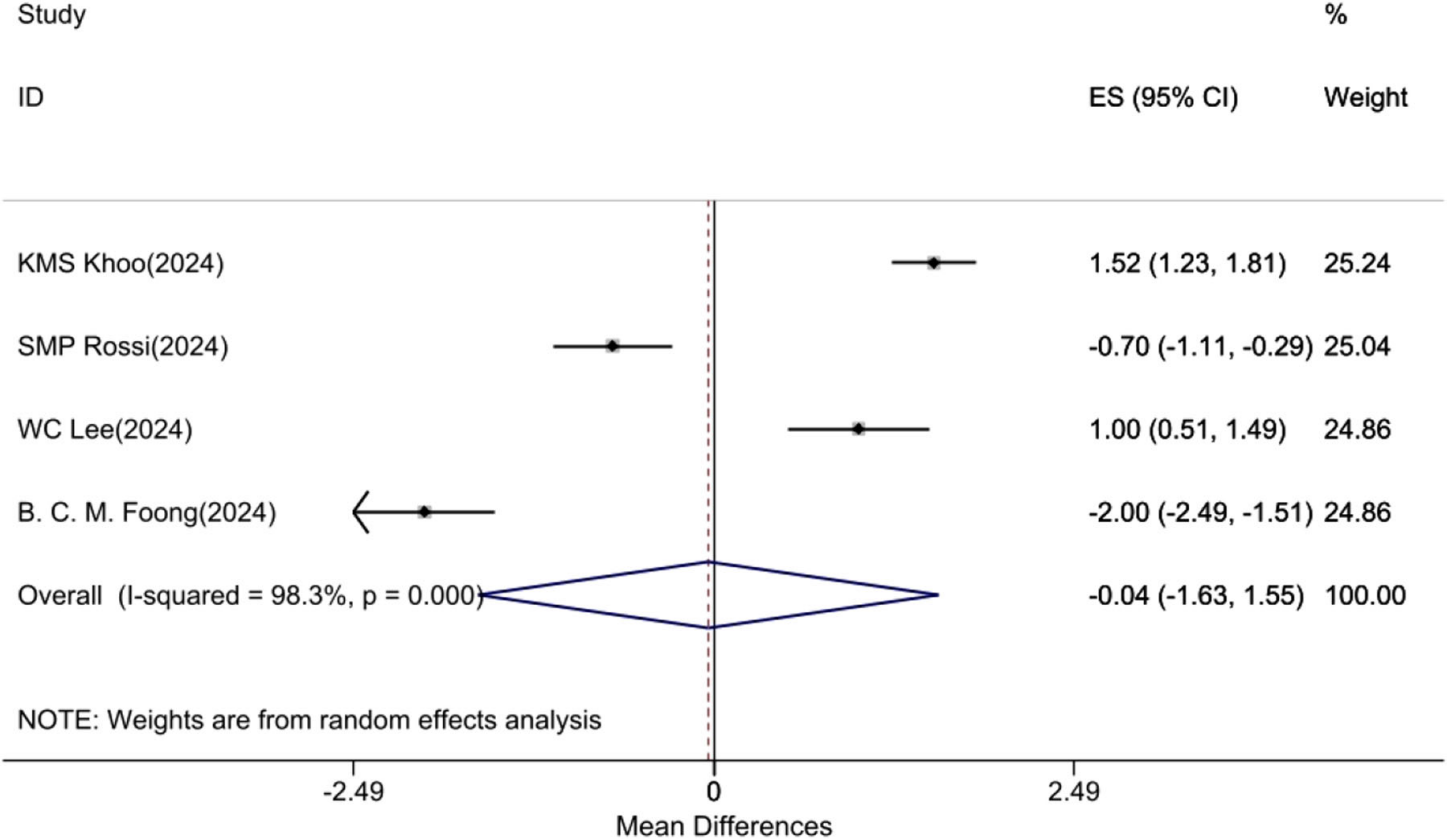
Difference in mean postoperative OKS between PR and PS groups after 12 months. CI, confidence intervals; OKS, Oxford Knee Score; PR, PCL retaining; PS, PCL sacrificing.

The evaluation of OKS scores at 3 and 12 months involved three and four studies, respectively [14, 27, 37, 58]. At 3 months, the pooled mean difference showed no significant difference between the PR and PS groups (0.17; 95% CI: –1 to 1.33, *I* ^2^ = 0%, *τ* ^2 ^= 0.11) (Figure 4). Similarly, at 12 months, the difference in mean OKS scores was not statistically significant (Mean Difference: –0.04; 95% CI: –1.63 to 1.55, *I* ^2^ = 98.3%, *τ* ^2 ^= 2.55) (Figure 5). The FJS score was reported in only one study [58], where the mean values for both groups were similar.

##### ROM

**Figure 6:**
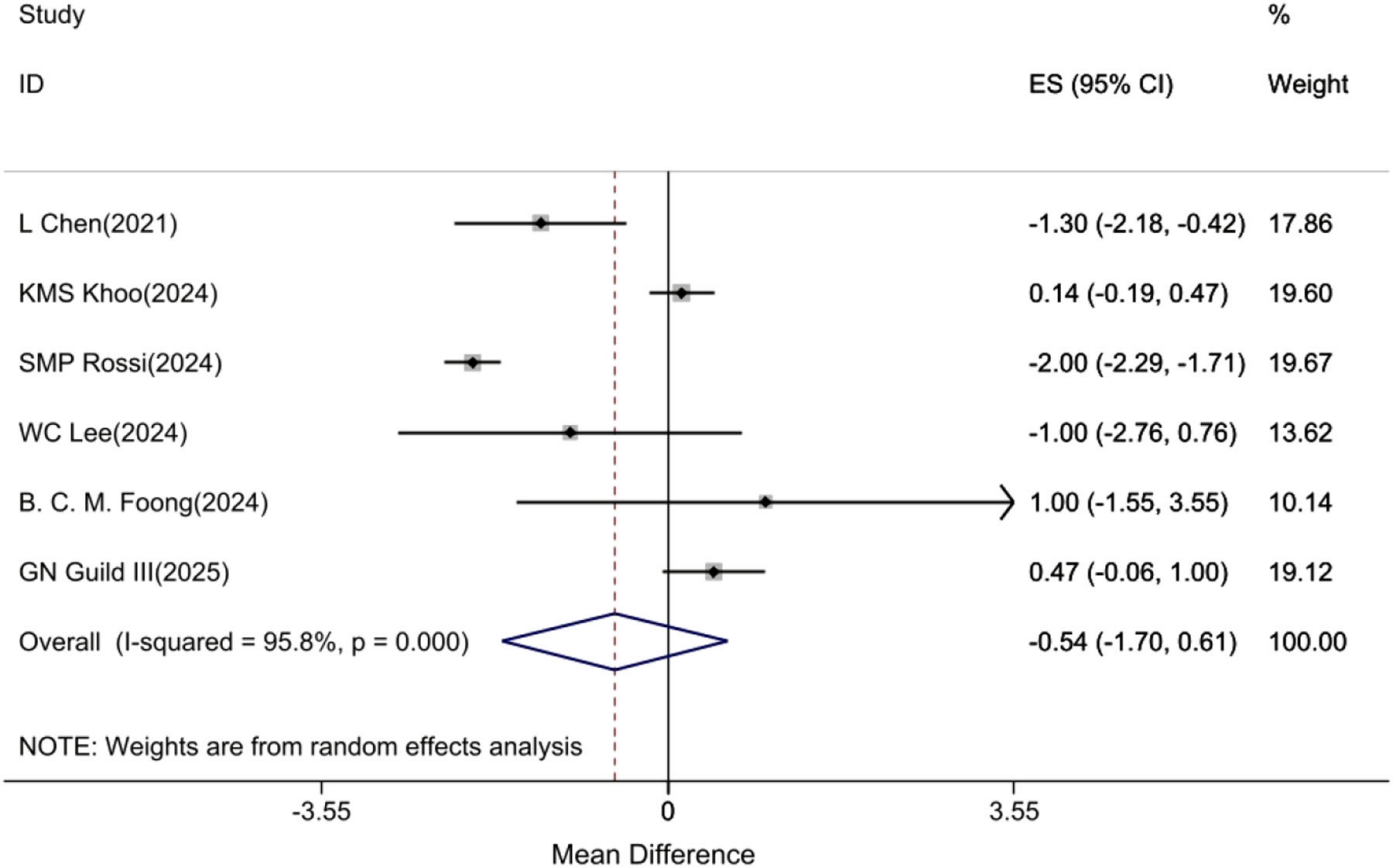
Difference in mean postoperative ROM score between PR and PS groups. CI, confidence intervals; PR, PCL retaining; PS, PCL sacrificing; ROM, range of motion.

The pooled analysis of ROM across six studies revealed no significant difference between groups (Mean Difference: –0.054; 95% CI: –1.7 to 0.61, *I* ^2^ = 95.8%, *τ* ^2 ^= 1.03) (Figure 6). The pooled estimate of ROM should be interpreted with caution due to considerable heterogeneity between studies.

### Complication

Only two studies reported the incidence of postoperative complications separately for the groups, and the estimates of the two studies suggest that the incidence of complications in the two groups may be similar. Guild III et al. [21] showed that at 3 months after surgery, the incidence of complications in the PR and PS groups was 30 (12.2%) and 28 (20.8%), respectively, and this difference was not statistically significant (*p* = 0.12). Complications in the PR group included readmission event (6), fall (3), manipulation under anesthesia (MUA) (2), Medical complication rate (9), Surgical complication rate (6), DVT (1), periprosthetic joint infection (PJI) (2), Periprosthetic fracture (1) and in the PS group included readmission event (5), Medical complication rate(7), fall (2), Surgical complication rate (7), DVT (3), MUA (1), PJI (1) and wound infection (2). Rossi et al. [58] reported two cases (one case per group) of wound infection one year after surgery.

#### Sensitivity and subgroup analysis

Sensitivity analysis was conducted to evaluate the impact of individual studies on overall outcomes (Supporting Information: Figure S1). The results of the sensitivity analysis showed that excluding studies with sample sizes of less than 50 patients in each group had the highest effect on the KSS and OKS scores. Heterogeneity for KSS and OKS was acceptable at both follow‐up intervals. Subgroup analysis revealed that the mean KSS and OKS scores differed significantly between the 3‐ and 12‐month follow‐up (Supporting Information: Figures S2 and S3).

### Publication bias

**Figure 7:**
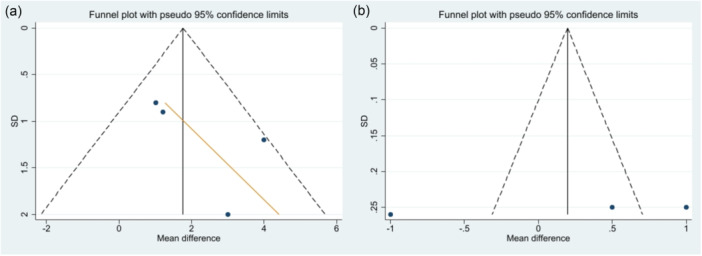
Bias publication assessment in the funnel plot for KSS (Graph A) and OKS (Graph B). KSS, knee society score; OKS, Oxford society score.

The Egger test results suggested no publication bias in KSS (*t* = 2.62, 95% CI: –5.8 to 11.3, *p* = 0.31) or OKS (*t* = –1.7, 95% CI: –6.71 to 4.2, *p* = 0.55). The distribution of studies in both KSS and OKS outcomes was nearly symmetric, affirming the absence of publication bias (Figure 7).

## DISCUSSION

The main findings included similar mean KSS and OKS in the PR group compared with the PS group at 3 months postsurgery. At 12 months postsurgery, mean KSS scores were significantly higher in the PR group than in the PS group. Although the difference was statistically significant, it remained below the established MCID threshold of 5.3 points for the KSS. Therefore, this finding does not represent a clinically meaningful improvement. The effects of PR and PS on KSS and OKS scores were below the MCID. According to a recent study, TKA with MC prostheses, regardless of PCL status (PR or PS), is consistently associated with improved patient satisfaction and clinical outcomes. This improvement may be attributed to various factors, such as prosthesis design and preservation of the patellofemoral ‘third space’, all of which can affect post‐TKA outcomes [35, 40, 51]. Furthermore, studies have shown that using congruent inserts for TKA may lead to better clinical and functional outcomes [8, 25, 40, 51, 61]. Sun et al. [63] demonstrated that patellofemoral stress reduction and postoperative complications are greater with congruent inserts. MC prostheses also mimic near‐natural joint kinematics and are associated with better functional and satisfaction outcomes. Konno et al. [29] also reported similar results for TKA patients with MC.

No clinically important difference was observed in mean OKS between the two groups at 12 months postsurgery. This difference between the OKS and KSS scores at 12 months after surgery can be attributed to changes in the indicators themselves, the sensitivity of the measurements, the patients’ interpretation of the questions, and their expectations. Giesinger et al. [17] showed that the sensitivity and responsiveness of patients in different scoring scales, including FJS, OKS, WOMAC and KSS, differed. It is noteworthy that the sensitivity and responsiveness of the FJS scale completed by the surgeon was superior to the KSS completed by the patient. This difference is likely due to differences in knowledge, understanding and expectations between the surgeon and the patient about these assessment scales. In another study, Goh et al. [18] showed that OKS may be more sensitive and accurate in predicting outcomes, especially in the short term.

Although the 12‐month difference in KSS scores is statistically significant, the clinical significance of this finding remains debatable due to the limited number of studies included in this meta‐analysis (six studies), their retrospective nature, and the inherent high risk of confounding and selection bias. The statistical significance of the results could be affected by random error (sample size of patients examined in the included studies) and systematic errors (Bias) related to study design, including the lack of access to and assessment of the effect of a number of confounders and key variables (due to the retrospective nature of the study), the assessment of functional indicators with patient self‐report scales, and the use of subjective measures that may have influenced the study results.

Becker et al. [5] presented normative data on distractor forces in tension‐controlled TKA in a meta‐analysis. They analysed 116 studies and reported mean forces of 149.9 N at 0° and 139.5 N at 90° of flexion, which were consistent across natural, anatomical and computational models. These results underscore the significance of standardised ligament tension and indicate that PCL management should focus on achieving optimal balance rather than merely preserving or sacrificing the ligament. Nevertheless, the potential benefits of personalised distractor forces for gap preparation remain uncertain. This uncertainty aligns with observations that patient‐specific factors, such as coronal plane alignment of the knee (CPAK) classification and base deformity, may affect the selection of optimal PCL management strategies.

Therefore, the design of more prospective studies with objective measures could be useful in assessing the long‐term effects of this difference on patient satisfaction, functional capacity and daily activities.

The incidence of complications was nearly identical in both groups; however, only two studies have assessed the incidence rate so far, and future research may yield different results as more studies are conducted in this area.

It should be noted that reporting only the mean and standard deviation (SD) of knee deformity does not include patients who are +1 or +2 SDs from the mean (i.e., those with more severe deformities). These patients are ‘out of the range’ with unique anatomy that is not represented by the population averages [62]. This problem is especially true when the surgeons use MA because in an important varus deformity, executing a MA alignment strategy will result in important soft tissue releases that can even result in medial instability in extension and/or flexion and lateral instability in flexion. When surgeons use a MA strategy in a patient with significant varus deformity (i.e., >2 SDs from the mean), they attempt to bring the knee to a neutral position of 0 degrees [6, 13, 60, 62, 64]. However, many of these patients have intrinsic varus—a natural, anatomical alignment that is not intended to be neutral. As a result, forcing a severely varus knee into a neutral position requires extensive soft tissue release on the medial side. This invasive release can lead to medial instability in extension and/or flexion and lateral instability in flexion [6]. These results suggest that surgeons should go beyond reporting mean/SD and use patient‐specific measurements such as arithmetic HKA (aHKA) and CPAK classification, and the goal should be personalised alignment based on the patient's natural anatomy, rather than a one‐size‐fits‐all, neutral mechanical target [13].

In a meta‐analysis, Vermo et al. [67] evaluated the impact of PR versus PS on functional outcomes and complication rates in patients undergoing medial pivot TKA. Their findings indicated that there was no significant difference in functional outcomes, complication rates or revision rates between PR and PS during TKA, which aligns with the results of our study. Additionally, Movassaghi et al. [41] conducted a review that demonstrated similar clinical and functional outcomes in PR and PS groups. Romano et al. [57], in a review of 39 articles including 6143 total knee replacements, showed that both MP and MC inserts showed good and similar results. They also found that clinical outcomes were similar between PR and PS, based on the limited results of studies that evaluated the role of the PCL in MP and MC inserts. The differences in outcomes among various studies may stem from variations in follow‐up periods, sample sizes, study methodology and the demographic characteristics of the participants. Studies have shown that older age can be associated with slower recovery after TKA. The majority of studies included in this meta‐analysis (four studies) were conducted in Asian countries. Studies have shown that functional outcomes after orthopaedic surgeries can be influenced by factors related to the geographic region, including race, socioeconomic characteristics, culture and lifestyle [16, 24, 32, 35, 65]. The geographic bias (predominantly Asian studies) has a potential impact on the generalisability of results to other populations with differing anatomy, activity demands or cultural expectations.

Heterogeneity was significant for a number of outcomes. The small number of included studies (*n* = 6) limited the power of our analysis and prevented robust subgroup analyses. However, some of the significant heterogeneity between studies for a number of outcomes could be explained by variation in follow‐up duration, quality of included studies, differences in time of outcome assessment (postoperative period), access to postoperative rehabilitation, outcome instruments (patient self‐report instruments), geographical variation and study population with different demographic characteristics patients (age, BMI and sexual distribution). Access to postsurgical rehabilitation services can be significantly associated with improved functional outcomes after TKA [30, 47]. Studies have shown that demographic characteristics, including older age, higher BMI and female gender were associated with poorer functional outcomes after TKA [3, 15, 33, 34, 55].

Due to the lack of reporting in the primary studies, we were unable to assess outcomes based on several key variables, including baseline deformities (varus and valgus), demographic characteristics (BMI and age) and the presence of flexion contracture, which may have contributed to the differing results. Studies have shown that preoperative deformities may affect functional activities and patient satisfaction after TKA, and that outcomes differ in patients with varus and valgus [11, 46, 56, 66]. Nishitani et al. [46] showed that functional activities and patient satisfaction were lower for patients with valgus knees than for patients with varus knees. MacDessi et al. [39] demonstrated by examining the utility of CPAK classification in predicting preoperative soft tissue balance and comparing KA technique with MA, they showed that when optimising soft tissue balance is a priority, CPAK classification can predict which knee phenotypes may benefit most from KA, and the choice of alignment technique should be patient‐centred.

The findings of this review suggest that surgeons can base PCL management decisions in medial axis TKA on surgical technique preferences, patient anatomy and intraoperative findings, rather than expecting significant functional differences.

Given the low to moderate certainty of the evidence as determined by the GRADE assessment, there is a need for high‐quality evidence to reach definitive conclusions. The majority of studies included in this systematic review were not Level I studies, which is a limitation; prospective Level I studies are therefore recommended to shed more light on this issue.

Our study had strengths and weaknesses that should be noted. Although the 12‐month difference in KSS scores is statistically significant, the clinical significance of this finding remains controversial due to random error (small sample sizes in the included studies) and systematic error (follow‐up and use of subjective patient‐reported measures) that may have influenced the study results. Due to a lack of reporting in the original studies, we were unable to estimate the effect of several key variables, such as patient satisfaction, quality of life, deformities (varus and valgus), demographic characteristics (BMI and age), the presence of flexion contracture, CPAK classification and kinematic outcome measures, such as prosthesis stability and survival, on the overall estimate. The inherent high risk of confounding and selection bias due to the exclusive inclusion of nonrandomised, retrospective studies and how this likely lowers the certainty of evidence (linking to the GRADE assessment), is a major limitation. Although we performed sensitivity and subgroup analyses, and the degree of heterogeneity was acceptable, the small number of included studies (*n* = 6) limited the power of our analysis and prevented robust subgroup analyses. Variation in timing and measurement methods for outcomes (patient self‐report instruments), limited geographical diversity and insufficient data on several outcomes were other limitations of this systematic review. The geographic bias (predominantly Asian studies) and its potential impact on the generalisability of results to other populations with differing anatomy, activity demands or cultural expectations. We only included studies published in English, as the results may be affected by publication language bias. Therefore, these results may differ across populations, and caution should be exercised when generalising this study's results to other populations. The clinical heterogeneity introduced by aggregating studies with different surgical techniques, surgeon preferences and potentially different MC implant generations may obscure true effects. The short‐ to mid‐term follow‐up of most included studies precludes any conclusions about long‐term outcomes. The evaluation of the effect of PR versus PS on functional outcomes in MC TKA in a meta‐analysis was a key strength of this study.

The heterogeneity among the studies was significant for the KSS and ROM and low for the OKS. The pooled estimate of ROM should be interpreted with caution due to considerable heterogeneity between studies. A subgroup analysis by follow‐up period revealed that most of the variability across studies could be attributed to differences in follow‐up duration. However, numerous other factors, such as patient demographic characteristics, sample size, study quality, CPAK classification and geographic differences, may also contribute to this variability. Unfortunately, we were unable to estimate these factors due to the limited number of studies included (fewer than 10). Fortunately, the majority of the studies were of moderate quality and demonstrated moderate certainty in their evidence. As the review includes exclusively retrospective cohort studies and no randomised controlled trials, the overall level of evidence should be clearly stated as Level IV to allow appropriate interpretation of the findings.

Given the low to moderate certainty of the evidence, there is a need for high‐quality evidence to reach definitive conclusions. The majority of studies included in this systematic review were not Level I studies, which is a limitation; prospective Level I studies are therefore recommended to shed more light on this issue.

## CONCLUSIONS

Based on low‐ to moderate‐certainty evidence (Level IV), there appears to be no clinically meaningful difference in PR or PS during MC TKA in improving short‐term clinical outcomes. The mean KSS at 12 months was statistically significantly higher in the PR group; however, this difference did not meet the MCID threshold. Both PS and PR in MC TKA with MA yield comparable and acceptable clinical outcomes. The incidence of complications was assessed in only two studies, which reported similar rates in both groups. Based on low‐ to moderate‐certainty evidence, surgeons can make decisions about PCL management based on their preferences rather than outcomes. These results were conducted in populations with specific characteristics, and caution should be exercised in generalising the results to other populations.

## AUTHOR CONTRIBUTIONS

**Mansour Bahardoust**: Conceptualisation; methodology; validation; formal analysis; investigation; data curation; writing—original draft preparation; writing—review and editing; supervision; project administration. **Mehdi Mohammadpour**: Conceptualisation; methodology; validation; formal analysis; investigation; data curation; writing—original draft preparation; writing—review and editing; supervision; project administration. **Habib Gorgani**: Investigation; writing—review and editing; project administration. **Meisam Haghmoradi**: Writing—original draft preparation; writing—review and editing; project administration. **Sajad Fakoor**: Methodology; investigation; data curation; writing—original draft preparation; writing—review and editing; supervision; project administration.

## FUNDING

The authors have no funding to report.

## CONFLICT OF INTEREST STATEMENT

The authors declare no conflicts of interest.

## ETHICS STATEMENT

The authors have nothing to report.

## Supporting information

Supporting File 1

## Data Availability

Data supporting the findings of this study are available upon reasonable request from the corresponding author.
